# Breath by Breath: Navigating the Landscape of Exhaled Breath in Clinical Research and Prospects for the Future

**DOI:** 10.1155/jamc/9959007

**Published:** 2026-05-06

**Authors:** Nisha Parikh, Hetansh Rao, Akshada Dabhade, Vishwa Patel, Vikalp Shroff, Deep Prajapati, Palak Parikh, Ketan Ranch, B. Mahalakshmi, Shery Jacob, Sai H. S. Boddu

**Affiliations:** ^1^ Department of Pharmaceutical Chemistry and Quality Assurance, L. M. College of Pharmacy, Navrangpura, Ahmedabad, 380009, Gujarat, India, lmcp.in; ^2^ Department of Pharmacology and Pharmacy Practice, L. M. College of Pharmacy, Navrangpura, Ahmedabad, 380009, Gujarat, India, lmcp.in; ^3^ Department of Pharmaceutics, L. M. College of Pharmacy, Navrangpura, Ahmedabad, 380009, Gujarat, India, lmcp.in; ^4^ Department of Microbiology, Government Medical College & Hospital, Rajiv Gandhi Institute of Medical Sciences (RIMS), Kadapa, Andhra Pradesh, India, gmch.gov.in; ^5^ Department of Pharmaceutical Sciences, College of Pharmacy, Gulf Medical University, Ajman, UAE, gmu.ac.ae; ^6^ Department of Pharmaceutical Sciences, College of Pharmacy and Health Sciences, College of Pharmacy, Ajman University, P.O. Box 346, Ajman, UAE, ajman.ac.ae; ^7^ Center of Medical and Bio-Allied Health Sciences Research, Ajman University, P.O. Box 346, Ajman, UAE, ajman.ac.ae

**Keywords:** analytical techniques for VOCs, biomarkers, disease diagnostics, environmental exposure, exhaled breath analysis, noninvasive method, VOCs (volatile organic compounds)

## Abstract

Exhaled breath analysis (EBA) has evolved as a noninvasive diagnostic technique with enormous promise in healthcare. The development in this area is based on the concept that the breathalyzers employed to measure the alcohol concentration in the blood can be utilized to evaluate health by analyzing the intricate chemical makeup of exhaled breath, which includes various volatile organic compounds (VOCs). Breath analysis could be the driver of personalized medicine and used in early disease identification as it involves the detection of a variety of disease‐specific biomarkers. In diagnostics, EBA offers a noninvasive procedure of illness identification and monitoring. This approach allows for early detection, personalized treatment options, and therapeutic monitoring by detecting disease‐specific VOC biomarkers such as acetone in diabetes and ethane in lipid peroxidation. Also, EBA can measure environmental exposures such as air pollution or occupational dangers. By identifying VOCs from various sources, researchers can measure exposure levels and assess related health hazards. Advances in the analytical methods, including mass spectrometry, gas chromatography, and sensor arrays made it possible to quantify and identify VOCs in exhaled air with high sensitivity and precision. This review focuses on the biomarkers present in breath and their role in identifying diseases. It covers the essential compounds found in breath and their biochemical processes. Furthermore, an in‐depth examination of different methods for identifying biomarkers in breath and the factors influencing VOC concentrations, including age, storage conditions, external sources, and the difference between dead space air and alveolar air, has been discussed.

## 1. Introduction

The investigation of breath is an emerging field for scholars with ancient roots. Breath tests have been used since the early days of medicine. Over the years, various practices such as preconcentration techniques, analysis methods, and contemporary methods of sampling were developed. Human breath consists of N_2_ (78.04%), O_2_ [16%], CO_2_ [4%–5%], H_2_ [5%], inert gases [0.9%], and water vapor. In addition, organic and inorganic volatile organic compounds (VOCs), including C_3_H_6_O (0.3–1 ppm), C_2_H_6_O, C_5_H_8_ (∼105 ppb), C_2_H_6_ (0–10 ppb), CH_4_ (2–10 ppm), C_2_H_5_ [0–10 ppb], and N_2_O (1–20 ppb), NH_3_ (0.5–2 ppm), CO (0–6 ppm), and H_2_S (0–1.3 ppm) are also contained in the breath. Linus Pauling, one of the earliest pioneers of breath analysis, reported 250 substances in human breath using gas–liquid partition chromatography analysis. Novel analytical methods for breath testing were developed during the 1990s. These consist of proton‐transfer‐reaction mass spectrometers, selective ion flow tube mass spectrometry, laser spectroscopy, and ion mobility spectrometry (IMS). The latest analytical instruments have made the process of diagnosis robust [1, 2].

The use of olfaction to recognize and evaluate illnesses using VOCs in human breath is subjective [3, 4]. The aptness to take frequent measurements that are less painful or distressing for a person undergoing scrutinization and further the property of noninvasiveness have led to an uninterrupted curiosity in diagnosing VOCs in the breath. It is possible to sample breath even during surgery or in a critical care unit. Hence, simultaneously analyzing and sampling of breath are considered an alluring prospect. Breath VOCs can originate from three distinct entities: the host, the environment, and the microbiome, which refers to the bacteria that live in the mouth, lungs, and stomach. Although clinically, endogenous and microbiological VOCs are of significant concern, many VOCs detected in the air exhaled have origins outside [5, 6]. The habits of a person and the surrounding environment are responsible for the exogenously originated VOCs. VOCs originating from cleaning agents, personal hygiene items, plastics, fires, and industrial or transportation gas emissions are inhaled for a prolonged period and then breathed out of the body. Significant sources of VOCs include food choices, prescription drugs, and dietary supplements. During breath analysis, it is necessary to consider the immediate and recent environmental exposure. Endogenously generated VOCs encompass products from normal or pathological metabolic pathways involving high vapor pressure (both at body temperature and room temperature) and microbiome metabolism [7, 8]. They usually arise in the lungs or in various parts of the human body and reflect the metabolism of the entire organism. The primary metabolic pathways associated with endogenous VOCs are peroxidation of lipids, cytochrome p450 (CYP450)‐catalyzed reactions, oxidative stress, and liver enzymes. The characterization of VOCs exhaled in the breath and the association between oxidative stress and airway inflammation are done. Different biochemical reactions are harmonized with different external sources and biochemical reactions [9]. Using breath testing, it is possible to monitor exposure to many harmful substances (such tetrachloroethylene) or 9‐tetrahydrocannabinol after marijuana use. The “discovery” of several gas‐phase particles in breath, including both endogenous and exogenous chemicals, was made possible by employing gas chromatography (GC/MS) and conventional mass spectrometry techniques [10].

EBA is at the forefront of innovation in clinical diagnosis and environmental exposure assessment. Its noninvasive nature, along with progression in analytical techniques and machine learning, has moved the discipline forward, allowing for early illness identification, tailored treatment tactics, and complete environmental health monitoring. Furthermore, integration with machine learning algorithms allows for pattern detection and biomarker development, which improves the diagnostic capabilities of exhaled breath study. Researchers have uncovered much information by analyzing the complex composition of exhaled breath, which has enormous potential for improving public health outcomes and guiding regulatory actions. As technology advances, EBA will play an important part in determining the future of healthcare and environmental science [11]. This review highlights the identification of biomarkers in breath and their application in diagnosing illnesses. This review addresses the compounds that are crucial to human breath and their metabolic pathways. In addition, the review includes a thorough discussion on various techniques used detecting biomarkers in breath and parameters affecting VOC levels such as age, storage conditions, exogenous origin, and dead space air as opposed to alveolar breath.

## 2. Clinically Relevant Analytes in Exhaled Breath

Exhaled breath is a complex mixture that mainly includes nitrogen (N_2_), oxygen (O_2_), carbon dioxide (CO_2_), water vapor, inert gases, and VOCs with molecular weights typically below 500 Da. These compounds are exchanged in the alveoli—the lungs’ functional units—and may also originate from the blood, saliva, skin, feces, and milk. Thus, breath can serve as a dynamic, noninvasive biomarker‐rich medium that reflects metabolic, infectious, and inflammatory states of the human body [12].

Over 3000 VOCs have been detected in human breath, with over 200 consistently present in normal individuals. These include both volatile and nonvolatile compounds such as hydrocarbons, alcohols, ketones, aldehydes, esters, cytokines, leukotrienes, isoprostanes, hydrogen peroxide (H_2_O_2_), and small inorganic gases like NO, NH_3_, H_2_S, CH_4_, and CO_2_. Among these, a select group of analytes—acetone, aldehydes, nitric oxide (NO), isoprene, ammonia (NH_3_), hydrogen sulphide (H_2_S), methane, ethane, and pentane—has emerged as especially clinically relevant for diagnosing a wide spectrum of diseases [13]. Table 1 contains detailed information about the diseases and their identification using biomarkers in breath.

**TABLE 1:** Diseases and their identification using biomarkers in breath.

No.	Disease	Biomarker (VOC)	Analytical technique	Data analysis	Ref.
1.	Cancer				
	Lung cancer	Acetone, isoprene, ethanol, propanol	GC/MS	ANN	[14]
		Formaldehyde, isopropanol	PTR‐MS	DA	[15]
		Acetone, methyl ethyl ketone, N‐propanol	GC/MS	*t* test, *U* test	[16]
		1‐Octene, 2‐methyl pentane, hexanone, 3‐heptanone, styrene, trimethyl hexane	SPME‐GC/MS	DFA as statistical pattern recognition	[17]
	Breast cancer	Nonane	GC/MS	DA	[18]
		2‐Propanal		FL function	[19]
		Cyclopropane		MC, multivariate WDA	[20]
		Hexanal, heptanal		Binary LR	[21]
	Prostate cancer	Toluene; 2,3,4‐trimethyl decane; p‐xylene; 2,2‐dimethyldecane	Portable E‐nose	PCA	[22]
	Liver cancer	3‐Hydroxybutan‐2‐one, decane	GC/MS	Mann–Whitney *U* test	[23]
	Ovarian cancer	Decanal, nonanal, hexadecane, styrene	GC/MS	DCA	[24]
		Pentanoic acid, hexanoic acid, phenol	SIFT‐MS	*U* test	
	Colorectal cancer	Ethyl alcohol	GC/MS, prototype E‐nose	DFA	[25]
		Propanenitrile, hendacane			
	Gastric cancer	2‐Propenenitrile, furfural, butoxyethanol, hexadecane, 4‐methyl octane, butanone	GC/MS, nanoarray sensor	DFA	[26]

2.	Malignant mesothelioma	Cyclohexane, toluene, xylol, benzaldehyde, trimethylbenzene, dipentene	Cyranose 320	CDA, PCA	[27]

3.	Diabetes	Acetone	E‐Nose	ANN, PCA	[28]

4.	Heart disorders				
	Congestive heart failure (CHF)	Isoprene plasma	GC/MS	MWR test	[29]
	Ischemic heart disorder	Malondialdehyde, propane, isoprene	HPLC	Fisher’s exact test	[29]
	Ischemic heart disorder	Pentane	SPME‐GC	*U* test	[30]

5.	Liver disease				
	Hemodialysis	Isoprene	GC	*t* test	[31]
	Ischemic liver disease	Ethane	GC	—	[32]
	Liver transplant	Carbonyl sulphide, dimethyl sulphide, dimethyl disulphide	GC	MR, MLR	[33]

6.	Tuberculosis	Cyclohexane, 1,3‐dimethyl‐ trans‐benzene, 2‐butanone, naphthalene	GC/MS	FL	[34]

7.	Cystic fibrosis	Benzothiazole, hydroxyoctanoic acid	SESI‐HRMS	Mann–Whitney *U* test	[35]

8.	Asthma	Nitrogen oxide	Cyranose 320	PCN, ANN	[36]

9.	Chronic obstructive pulmonary disease	Nitrogen oxide, pentane, hydrogen peroxide	Cyranose 320	LDA	[37]

10.	Acute respiratory distress syndrome (ARDS)	1‐Octane, isoprene, ethylene, ethyl aldehyde	GC/MS	PCA, LDA	[38–41]

11.	Pneumonia	Bacterial metabolites	DiagNose	PCR, LR	[42]

12.	Neurodegenerative Disorder				
	Parkinson’s disease	Aldehydes	NMVS	DFA	[43]
	Alzheimer	Styrene	NMVS	DFA	

13.	Renal disease	Dimethylamine, trimethylamine, ammonia	E‐Nose	PCA	[44]

14.	Sleep apnea	Pentene, isoprene, butanol	GC/MS	—	[45, 46]
		Isoprene	SESI‐MS	—	[47–49]

15.	Smoking	Acetaldehyde. propionaldehyde, acetone	GC	*t* test	[50, 51]

16.	SARS‐CoV‐2/COVID‐19	Ethanol, octanol, acetone, butanone, methanol	GC/MS	PCA, LDA	[52]

**FIGURE 1:**
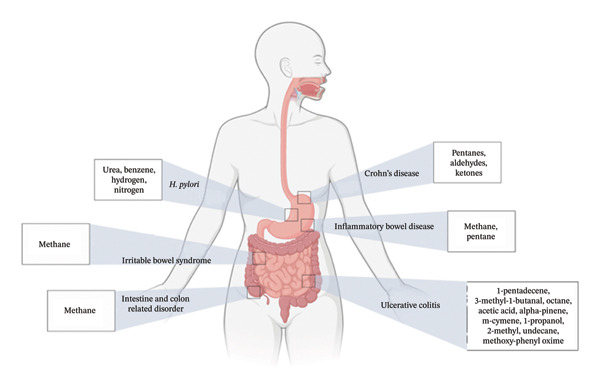
GI tract conditions and their biomarkers.

**FIGURE 2:**
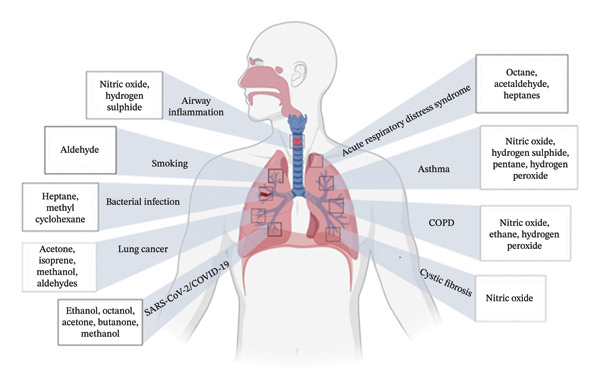
Respiratory conditions and their biomarkers.

**FIGURE 3:**
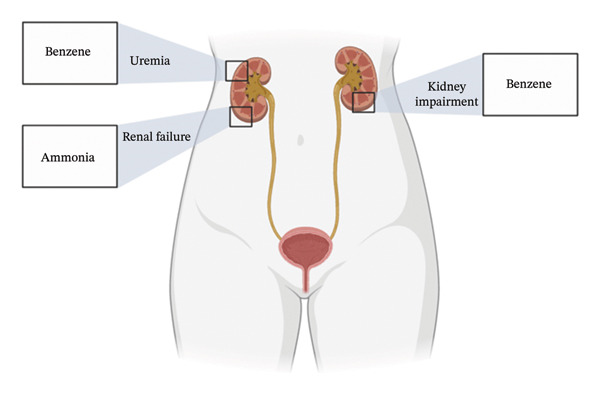
Renal conditions and their biomarkers.

**FIGURE 4:**
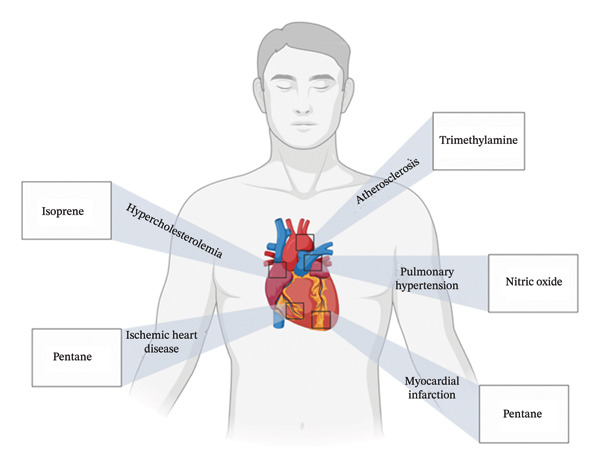
Heart conditions and their respective biomarkers.

**FIGURE 5:**
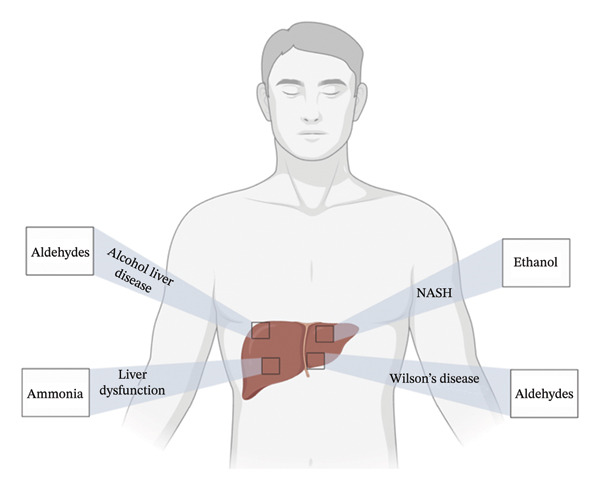
Hepatic conditions and their biomarkers.

**FIGURE 6:**
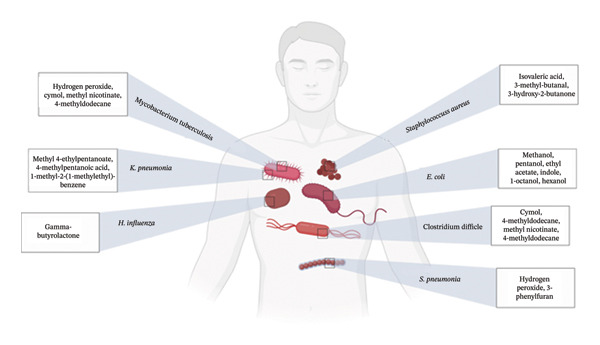
Various microorganisms and their biomarkers.

Figures 1, 2, 3, 4, 5, and 6 illustrate how these analytes are affected by or reflect dysfunction across various organ systems including the gastrointestinal tract (e.g., *H. pylori* infection), lungs (e.g., COPD and asthma), kidneys (e.g., uremia), cardiovascular system (e.g., trimethylamine and isoprene), and liver (e.g., oxidative stress).

Below, the most important and interesting biomarkers are described in detail.

### 2.1. Acetone

Acetone is one of the most prominent VOCs in exhaled breath. It is produced during β‐oxidation of fatty acids and ketone metabolism in the liver. Elevated levels of breath acetone are commonly observed during fasting, malnutrition, intense physical activity, diabetic ketoacidosis, or ketogenic diets. Concentrations can rise significantly in individuals with poorly controlled diabetes, where normal levels (∼0.9 ppm) may become several‐fold higher.

### 2.2. Aldehydes

Aldehydes are typically generated during lipid peroxidation (LPO) of polyunsaturated fatty acids (PUFAs) triggered by oxidative stress and free radicals. Elevated breath aldehydes are found in patients with Wilson’s syndrome, hemochromatosis, coronary artery disease, liver cancer, pediatric cancer, fatty liver disease, smoking‐related disorders, and diabetes. Due to their direct correlation with cellular damage and malignancy, aldehydes are especially useful in lung cancer diagnostics.

### 2.3. NO

NO is a key exhaled biomarker for airway inflammation and is particularly relevant in diseases such as asthma, COPD (chronic obstructive pulmonary disease), and cystic fibrosis. Its measurement as fractional exhaled NO (FeNO) is a standard noninvasive tool to assess eosinophilic inflammation. Elevated FeNO levels (> 50 ppb) suggest active inflammation, especially due to inducible NO synthase (iNOS2) activation. Guidelines by the American Thoracic Society recommend interpreting FeNO values with clinical cut‐offs. The development of portable analyzers like NIOX MINO has facilitated real‐time diagnosis and asthma management.

### 2.4. Isoprene

Isoprene is a hydrocarbon resulting from cholesterol biosynthesis and is detected in healthy breath at ∼105 ppb. Its levels rise with age, peaking by age 25. Along with acetone, isoprene is gaining attention for its role in the early detection of metabolic disorders such as diabetes.

### 2.5. Methane, Ethane, and Pentane

These hydrocarbons reflect microbial activity and oxidative stress. Methane is not typically found in human breath unless gut methanogens increase its systemic presence. Ethane and pentane arise from lipid peroxidation, with elevated levels linked to oxidative stress, inflammatory bowel disease, ulcerative colitis, vitamin E deficiency, arthritis, asthma, COPD, and breast cancer.

### 2.6. NH_3_

NH_3_ in breath reflects liver and kidney function. It is a by‐product of protein and nucleic acid metabolism, detoxified in the liver through the urea cycle. Elevated breath NH_3_ (normal range: 100–250 ppb) is observed in hepatic encephalopathy, liver disease, renal failure, and type II dementia. The foundational detection of amines in human breath was confirmed in 1977.

### 2.7. H_2_S

H_2_Sis a sulfur‐containing gas with a characteristic odor. It is produced during incomplete metabolism of methionine and has been implicated in liver and renal disorders. The normal breath concentration is 8–16 ppb. H_2_S has been identified as a useful biomarker in diseases such as asthma, oral and dental health disorders, and systemic airway inflammation.

Detailed explanation is mentioned in Table 2.

**TABLE 2:** Authorized FDA breath tests.

Sr. no.	Analyte/marker	Test name	Approval status	Clinical application	Reference
1	Fractional exhaled NO	Breath Test for FeNO (e.g. NIOX)	FDA‐approved	Diagnosing and tracking asthma	[53]
2	^13^CO_2_	^13^C breath test with aminopyrines	Research/clinical use	Analysis of liver function	
3	NH_3_	Test for ammonia breath	Investigational	Assessment of renal and hepatic functions	
4	3‐Heptanone	Test of valproate metabolism	Research only	Anticonvulsant therapeutic medication monitoring	
5	^13^CO_2_	Test of ^13^C urea breath	FDA‐approved	Identification of *Helicobacter pylori* infection	
6	^13^CO_2_	^13^C methacetin breath examination	CE‐marked, clinical use	Function of liver microsomes (CYP1A2 activity)	

7	H_2_/CH_4_	Test for hydrogen breath	Widely used clinically	Lactose/fructose malabsorption, SIBO	[54]
8	Trimethylamine	Identification of trimethylamines	Experimental	Chronic kidney disease with trimethylaminuria	

## 3. Analytical Techniques for Studying Exhaled Breath VOCs

Current breath VOC research leverages a diverse toolbox of analytical approaches. While ^13C‐labeled substrate tests offer precise metabolic tracing, other methods provide high‐throughput, sensitive, or real‐time capabilities.

### 3.1. Analytical Approaches for Breath VOC Detection

A variety of analytical platforms have been developed to detect and characterize VOCs in exhaled breath. These methods differ in terms of sensitivity, temporal resolution, portability, and suitability for targeted or untargeted analysis. Some approaches are well established and primarily laboratory‐based, while others allow for real‐time or point‐of‐care breath analysis. The following sections summarize the most commonly used techniques, with a focus on their integration into breathomics workflows.

#### 3.1.1. Gas Chromatography–Mass Spectrometry (GC‐MS)

Because of its high sensitivity, reproducibility, and robust compound identification using spectral libraries and retention indices, GC‐MS remains the standard for breath VOC analysis. Because breath VOCs occur at trace (ppb‐ppt) levels, sample preconcentration is usually necessary. Breath‐compatible strategies such as solid‐phase microextraction (SPME), thermal desorption (TD) with sorbent tubes, and needle‐trap devices (NTDs) have been successfully integrated into GC‐MS workflows, resulting in efficient VOC enrichment and better quantitative performance. GC‐MS, despite its limitations in offline analysis and longer turnaround times, remains indispensable for biomarker discovery, method validation, and VOC identity confirmation, as well as serving as a benchmark for emerging breath analysis technologies.

#### 3.1.2. Selected‐Ion Flow‐Tube Mass Spectrometry (SIFT‐MS)

SIFT‐MS is a soft‐ionization technique that allows for real‐time, quantitative analysis of breath VOCs without the need for preconcentration or chromatographic separation. SIFT‐MS uses well‐characterized reagent ions (H_3_O^+^, NO^+^, and O_2_^+^) to reduce fragmentation and simplify spectral interpretation. This allows for accurate quantification of multiple VOCs at ppb–ppt levels. Its rapid response and low sample preparation requirements make it ideal for clinical breath monitoring and longitudinal studies.

#### 3.1.3. Proton‐Transfer‐Reaction Mass Spectrometry (PTR‐MS)

PTR‐MS enables online, high‐temporal‐resolution monitoring of VOCs in exhaled breath, with detection limits in the low ppt range and response times in the milliseconds. The technique uses proton‐transfer reactions with H_3_O^+^ ions and does not require sample preparation. PTR‐MS has been widely used in clinical breathomics, metabolic profiling, and environmental exposure assessment, though its limited compound separation capability can make isomer discrimination difficult.

#### 3.1.4. Secondary Electrospray Ionization Mass Spectrometry (SESI‐MS)

SESI‐MS allows for the direct, real‐time detection of VOCs and semivolatile metabolites from breath with high sensitivity and temporal resolution. This technique has proven particularly useful for tracking rapid metabolic changes, as well as distinguishing pathogen‐specific breath signatures in vivo, such as *Staphylococcus aureus* and *Pseudomonas aeruginosa*.

#### 3.1.5. IMS and GC‐IMS

IMS, either as a standalone method or in combination with gas chromatography (GC‐IMS), separates ionized VOCs based on their mobility in an electric field. These systems are ideal for near‐patient testing due to their rapid analysis, compact instrumentation, and high sensitivity. Pilot studies have shown that IMS‐based approaches have the potential to detect lung cancer and airway infections [10, 55].

#### 3.1.6. Electronic Noses (E‐Noses) and Sensor Arrays

Instead of detecting individual compounds, E‐noses detect VOC patterns using arrays of cross‐reactive sensors. When combined with advanced machine learning algorithms such as drift compensation and domain adaptation techniques, E‐noses allow for rapid and low‐cost breath profiling. Despite their lack of molecular specificity, their portability and ease of use make them promising tools for point‐of‐care screening and large‐scale population research [56].

#### 3.1.7. Optical and Laser Spectroscopy Techniques

Optical techniques such as cavity ring‐down spectroscopy (CRDS), off‐axis cavity‐enhanced absorption spectroscopy (OF‐CEAS), and photoacoustic spectroscopy—including quartz‐enhanced photoacoustic spectroscopy (QEPAS)—provide highly selective detection of specific breath biomarkers (e.g., acetone, methane, and hydrogen sulfide) with fast response times and ppb–ppt sensitivity. These methods are ideal for targeted breath analysis and real‐time monitoring applications [10, 57, 58].

### 3.2. ^13^C‐Labeled Breath Tests

These techniques use administration of labeled substrates (e.g., ^13^C‐methacetin, ^13^C‐aminopyrine, and ^13^C‐urea) to monitor metabolic function—particularly hepatic microsomal activity and *Helicobacter pylori* infection. Common metabolized products like ^13^CO_2_ and ^13^CO_2_ kinetics serve as noninvasive liver or gastric biomarkers plus monitor NH_3_ and trimethylamine in renal function and FeNO for respiratory inflammation.

## 4. Breath Biomarkers Detecting Techniques and Biosensors

Breath biomarkers or VOCs in exhaled breath can reveal vital information about a variety of physiological and pathological disorders. The identification of these biomarkers has received interest for its noninvasive nature and prospective uses in medical diagnosis. Analytical methods are techniques used to determine the composition or properties of a sample. These methods are quite vital in various fields such as chemistry, biochemistry, environmental science, and materials science. Analytical methods are known for selectivity, sensitivity, accuracy, precision, linearity, robustness, and LOD and LOQ features that make them easy to use. Some of recent techniques used in the detection of trace gas contents in exhaled breath are listed in Table 3. The choice of technique depends on factors such as specific biomarkers of interest, the required sensitivity and specificity, cost considerations, and the intended application (e.g., research, clinical diagnosis, and monitoring). Ongoing research is continually improving existing techniques and exploring new methods for breath biomarker detection.

**TABLE 3:** List of techniques used for exhaled breath analysis and detecting breath biomarkers.

Technique	Principle	Limit of detection	Mode of operation	Advantages	Disadvantages	Ref.
GC/MS	Isolating and determining compounds by mass spectrometry utilizing a chromatography	ppb and ppt	Offline	Highly sensitive and selective	Preconcentration steps are bulky, time‐consuming to sample, and necessitate standards and a skilled operator	[59–61]
GC‐GC/MS	Separation of analyte archive by passing it through two columns and detecting it by using MS	ppb	Offline	Better separation than conventional one	Expensive	[62–64]
GC/TOF MS	Identification of analyte using time‐of‐flight detector	ppb	Offline	Provide more stable result	Higher cost	[65, 66]
FT‐IR	Detecting the radiation passing through sensor	ppm	Online	Absorption spectra of many substances obtained quickly	Low sensitivity	[65, 67, 68–73]
QCL	Cascade electronic transition in injection region	—	Real‐time	Operate at room temperature	Operate only in mid‐IR region	[67–73]
DFB‐QCL	Laser emission due to interband transition	ppb	Real‐time	Fast response and sensitive	Permit the detection of only one marker gas	[73–75]
EC‐QCL	Providing single mode laser output and outer cavity containing a diffraction grating on a rotating platform	ppm	Online/real‐time		Poor sweep to sweep reproducibility	[75, 76]
SIFT‐MS	Quadruple evaluation of particles generated through reactive analytes and ionic particles.	ppb and ppt	Real‐time	Broad detection range ppt capacity	Less sensitive than PTR‐MS	[76–78]
PTR‐MS	MS evaluation of ionized molecules of goal analytes using H_3_O+	ppb and ppt	Online/real‐time	Investigation in real‐time	Require trained personnel	[76, 78, 79]
SESI‐MS	Generating charged particles for mass spectrometry detection	ppb	Online/real‐time	Multiple ionization modes are available, sensitive and reliable tool	Bulky compound cannot be identified and requires a trained operator	[55]
IMS	Ion separation according to analyte mobility in electrical field	ppb	Real‐time	High sensitivity and low cost per analysis with high‐speed data acquisition, no vacuum needed	Atmospheric vapor contamination and complex spectra	[77, 80]
GC/IMS	Separation of different ions while moving in the electric field in gas phase	ppb/ppt	—	Better selectivity	—	[80–83]
MCC‐IMS	Preseparation of breath in multicapillary column and then entering ionization chamber of IMS	—	Online	Cheaper and user friendly	Data need to be validated by using GC/MS	[84, 85]
LIF	Emitting the fluorescence when excitation happens at suitable laser wavelengths	ppb/ppt	Real‐time, online	Qualitative and quantitative analyses, small sample size required	—	[86–88]
LITES	Light causes vibration on quartz tuning fork which is converted into electrical signal by quartz tuning fork piezoelectric effect	ppb	Online	A photodetector with a full wavelength response range is not required	—	[88–90]
TDLAS	Absorption of laser at a particular wavelength is measured	ppm/ppb	Real‐time	Ability to identify and quantify VOC molecules with extremely high selectivity	Poor signal‐to‐noise ratios	[91–95]
LAPS	Exciting the sample gas using laser inside the spectrophone and measuring by a two‐channel power meter detector	ppb	Online	Ability of measuring a minimum concentration is 0.2 ppb of ethylene in nitrogen	Necessary to remove CO_2_ from breath	[96, 97]
CRDS	Measuring the decrease in the intensity of laser pulse because of absorption	ppb	—	Sensitivity of detecting ammonia is 50 ppb	Work only in a single frequency range at a time	[98, 99]
CEAS	Advanced version of the CRDS, principle based on uses of off‐axis adjustment of the optical cavity	ppb/ppt	Real‐time	Simpler, cost‐effective	Spectral range depends on the reflectivity index of the mirrors	[98, 100–105]
OF‐CEAS	Optical feedback is used to secure the laser within the cavity while scanning the wavelength with a comb at the resonant frequency due to molecular absorption	ppb	Real‐time data online measurement	Good spectral resolution and high signal‐to‐noise ratio	—	[106–110]
CEPAS	Measuring a signal produced by the energy relaxation of the excited molecules	ppm	—	High sensitivity	Use of a Michelson interferometer makes the system complex	[111–114]
QEPAS	Laser radiation absorption by gas molecules which cause the periodic heating of the molecular species	ppb	Online	Portable devices with low power consumption	Needs higher amount of sample gas volume and additional maintenance requirements	[115–120]
OFCS	Measuring frequencies of light from the invisible infrared and ultraviolet to visible region	ppm		Extreme sensitivity, high‐resolution spectroscopy	Main drawback is bulky and complicated	[121–124]
OA‐ICOS	Consists of two mirrors, where light bounces up for many times to provide long absorption path	ppb	Real‐time and continuous monitoring	High sensitivity and fast response	Main issue is fluctuation in spectral baseline	[125–131]
CW‐ICOS	Using two mirror optical cavities employing single mode tunable diode laser as injection light source	ppm/ppt	Real‐time	Stabilization of cavity is not required	Modulation of diode frequency is required	[131, 132]
SERS	Interaction between the radiation and molecular field	ppb	Real‐time	Low cost, good accuracy, and reproducibility		[132, 133]
FERS	Transmitted light passes through hollow fiber	ppm	Online	Simultaneous detection of disease marker is possible	Need to ensure constant pressure within fiber	[96]
E‐Nose	Olfactory receptors produce electric signals when interaction with odor receptors happens	ppb	Real‐time	Portable, low cost, and user friendly	Not useful for screening of multiple diseases	[134–138]
Colorimetric sensor	Electrical signal converts into a digital signal using analog‐to‐digital signals causing color change	ppb	—	High selectivity and sensitivity	Color changes accuracy depending on the time of exposure of the sensor to the analyte	[139–143]
Chemiluminescence analyzer	Measuring the intensity of emitted light from excited analyte molecule	ppb	Online	Overly sensitive and standard technique for measurement of nitric oxide (NO)	System is costly and bulky	[143–146]

### 4.1. Exhaled Breath Condensate (EBC)

EBC is a relatively easy, noninvasive matrix that is produced by cooling and condensing exhaled air and is used to evaluate both volatile and nonvolatile biomarkers. EBC is defined by the European Respiratory Society (ERS) and American Thoracic Society (ATS)‐supported Task Force as a diluted solution that contains a variety of biomarkers with varying levels of chemical stability. For EBC collection, a number of commercial devices are currently on the market. During tidal breathing, EBC is typically collected over a predetermined period into a condenser composed of a chemically neutral substance. The ERS/ATS guidelines for EBC collection emphasize that higher expiratory flow rates decrease condensate collection efficiency, which dilutes the EBC sample that is collected. A variety of volatile and nonvolatile biomarkers, including cytokines, leukotrienes, oxidative stress markers, and molecular biomarkers, are present in EBC and provide important insights into lung inflammatory and neoplastic conditions. Depending on the kind of EBC collection device being utilized, the temperature range at which cooling occurs might vary from 0 to less than −20 degrees Celsius. It is advised to employ a saliva trap and nose clip. The exhaled air cools and condenses, creating a liquid sample from which a variety of chemicals can be identified. When exhaled humidified air condenses, a very simple matrix known as EBC is created, usually consisting of more than 99% water.

The main benefits of EBC sample collection are its total noninvasiveness, ease of use, and capacity to conduct several tests in almost any setting. Additional external fluid, which is usually supplied to the airways during procedures like bronchoalveolar lavage (BAL) or the delivery of drugs, is not required. Patients of all ages who are actively breathing and those on mechanical ventilation can undergo the EBC sample technique by connecting to the ventilator’s expiratory circuit. Thus far, no negative consequences of the EBC sample process have been reported in patients with lung diseases, including severe lung problems, in either the adult or pediatric populations. Furthermore, the stability of molecules like miRNAs and the noninvasiveness of EBC sample point to the possible application of these biomarkers as indicators for lung cancer and other chronic lung disorders.

Standardization in sample collection and interpretation is necessary for the acceptability of EBC analysis in clinical practice. To identify the low analyte concentration in EBC, more sensitive and focused analytical methods are required. Clinical diagnostics will be based on a database that contains typical physiological ranges for different EBC biomarkers, which should be created as a result of standardizing EBC procedures. The cost of a single test, which is mostly caused by the technical requirements of extremely sensitive testing methods, may also be a problem. This is particularly true when data regarding the biomarker profile or clusters are developed. The cost may also be a problem, not only regarding the collection and storage of EBC samples. Population studies that would enable the construction of trustworthy discriminant factors and reference points during analysis and interpretation are also severely lacking.

## 5. Evaluation of Exposure to VOCs

### 5.1. Overview of Breathomics and Gas Exchange

Breathomics refers to the knowledge of exhaled breath components such as CO_2_, O_2_, or NO and other GVOs (gas‐phase VOCs) like acetone, isoprene, NH_3_, and ethanol. Upon breathing, various gases, such as nitrogen (80%), oxygen (20%), and other gases (including 0.04% carbon dioxide), enter the lungs and reach the alveoli, the endpoint of the respiratory tract. In the alveoli, gas exchange occurs through diffusion of oxygen from the alveoli into the pulmonary capillaries and into the blood [147]. This process supplies the cells with energy by metabolizing sugars, forming CO_2_ as a waste, which goes back into the bloodstream and then to the lungs. Upon exhalation, CO_2_ containing breath is expelled, completing the cycle of gas exchange. Advantage and limitations of EBA are listed in Table 4 [148].

**TABLE 4:** Pros and cons of exhaled breath analysis.

Advantages	Limitations
Noninvasive, nonintrusive	Errors in sampling brought on by food intake and diet
Enables repeated sampling	Physician acceptance
Cheap if the appropriate method of collection is employed	Efficiency of collection (dilution of water vapor and dead space)
Hospital‐to‐home solutions potential	To be approved as sample for analysis
Personalized medication through analysis of breath prints	Data from various patient groups must be gathered

### 5.2. Exposure and Routes of Entry

The National Academy of the Sciences defines exposure as an event that occurs when a contaminant of a given concentration crosses natural human being barrier with the environment for a period and then stays there [15, 148, 149]. Thus, exposure needs the occurrence of two events at the same time: a pollutant concentration at a certain location and time and the presence of a person at that location and time. Chemicals from the environment enter the body by one or more of three routes: ingestion, inhalation, and dermal contact. The chemicals can be taken through the blood into the bloodstream or pass through the body unabsorbed and then straight out in the feces [150]. A study examined the benzene levels in the blood, and alveolar air of 168 male volunteers was determined within the age range of 20 to 58 years. The volunteers were grouped into four—hospital staff, blood donors, and chemical workers with and without exposure to benzene. The level of benzene in the air sacs was much higher in smokers compared to nonsmokers among both hospital employees and those not exposed to the environment, but this difference was not observed in blood donors or those not exposed at work. When looking at the three groups without any work‐related exposure, there was a strong link between the benzene levels in the air sacs and the benzene levels in the environment at the time of the tests, for both smokers (*r* = 0.636; *p* less than 0.001) and nonsmokers (*r* = 0.628; *p* less than 0.001). Similarly, in the same three groups and among those exposed at work, the benzene levels in the air sacs were closely related to the benzene levels in the blood [151]. While similar studies in the Europe and Asia have shown results that are comparable to the Total Exposure Assessment Methodology studies that were conducted in the United States, this shows that the results from the two regions are the same [152]. The far‐reaching common finding obtained from some studies on this is that the primary sources of exposure are small and close to the body, frequently observable.

### 5.3. Biomonitoring and Elimination via Breath

Those contaminants absorbed into the blood will undergo metabolism/excretion or retain in the body until equilibrium with the blood plasma concentration is reached or gently removed. Blood and urine are the primary matrices used for biomonitoring. In addition, expired air matrices, secretion, and excretion matrices of milk, sweat and saliva, and the storage matrices of adipose tissue and fat are also used [6, 153]. Persistent chemicals that are volatile in nature with short biological half‐life are eliminated in urine or expired air. Therefore, the breathing gas of exposed subjects is the analyte of choice for detection of organic volatile compounds.

### 5.4. Advantages of Breath Analysis Over Blood

Furthermore, while both blood and breath measurements can be employed to evaluate human body exposure to VOCs, the preferred and more sensitive method for determining the body burden of most VOCs is through measuring exhaled breath. VOCs enter the body primarily by inhalation and then travel across the breath–blood contact in the respiratory system. According to pharmacokinetic models, breathed air remains in the alveoli for an extended period, allowing VOCs and arterial blood to reach equilibrium. The partition coefficient, which determines the proportionate content of each VOC at the blood–breath interface, is essential for establishing equilibrium. Nevertheless, there is indication that the ratio between blood and breath increases as concentrations decrease to levels typical of environmental exposures in the parts per billion (ppb) range. Reliably calculated partition coefficient serves as a basis for the evaluation of blood concentration in arteries of breath analysis. Therefore, in combination with blood concentration measurement, a tissue distribution model provides a possibility to calculate the concentration in different body tissues [148, 154].

## 6. Parameters Affecting VOC Levels

Several parameters such as age, storage condition, exogenous condition, and dead space air as opposed to alveolar breath affect the VOC level. For instance, children tend to have a lower concentration of isoprene in their breath compared to adults. Isoprene concentration is higher during the puberty stage. Moreover, a few studies showed that NH_3_ concentration in the breath grows significantly with age. No doubt, the amounts of VOCs in the exhaled breath vary with the age, gender, food habits, and other miscellaneous factors [155]. There is an increase in the concentration of ethanol in the breath after intoxication. Likewise, breath has a different odor after eating onions, garlic, mint, and ice cream flavors. Even though no meaningful relationship was found yet, the constitution of the breath that is exhaled is affected if a woman is pregnant [155–158].

### 6.1. Storage Conditions

Storage conditions impact the breath analysis result; hence, direct analysis is preferred. However, direct analysis is not supported in all scenarios. So, care must be taken at every point to avoid loss of the components of the breath due to the influx of VOCs from the storage container, diffusion, and background emission of pollutants. Tedlar bags have gained popularity for breath storage. Glass vials (SPME), metal containers, micro‐packed absorbent traps, Nalophan containers, and Flexfoil packages are also preferred for breath storage [159].

Exogenous origin: both endogenous and exogenous origin VOCs are present, such as NO, NH_3_, and benzene. Exogenous VOCs could be either absorbed by the skin or inhaled through the lungs. Thus, a health condition would not always be reflected by the proportion of such VOCs in a breath expelled [160]. There is no correlation between blood VOC concentration and exhaled number of VOCs when inhaled chemical quantity surpasses 5% of exhaled volume [161].

### 6.2. Dead Space Air as Opposed to Alveolar Breath

The total air exhaled is the sum of the air that was inhaled (dead space air) and the alveolar air. VOCs that are extremely soluble and dilute are simpler to measure in exhaled breath. Detecting highly soluble volatile organic molecules like isoprene and acetone is challenging. The basic channel for the exchange of VOCs is the airway system, not the alveoli [162]. Soluble gases are taken up by the airway upon breathing. To get around the substantial concentration variations between the gas inhaled and the saturated solubility gases in the alveoli, an ingenious fractional blender was developed. A portion of the solubilized gas gets recreated in the mucus that covers our airways during exhale. The soluble gas is diluted as it enters the pulmonary system. Moreover, the amount of soluble gas released decreases with a rise in blood flow [163]. There may be a clear conclusion drawn from the study of the exhaled air if the breath is kept for ten seconds before being released. The more favored mode is breath exhaled via the mouth as opposed to air exhaled through the nose. Bacterial activity in the mouth, oral cavity, and airway produces VOCs that are exhaled together with mucus and saliva. It may be challenging to track health and recognize diseases based just on breath as a result.

## 7. Prospects

The experience from recent studies indicates that the breath condensate collection is gaining popularity in the scientific community. Breath can be noninvasively captured whenever necessary, notably during surgery or sleep, which forms the foundation for breath analysis. Melanoma cells release special chemical markers in blood and urine and this in turn releases volatile chemicals on the skin surface in quantities sufficient to allow early diagnosis. Trained canines are being efficiently used to detect VOCs as cancer indicators, particularly in lung cancer.

### 7.1. Cancer Identification Utilizing the Canine Sense of Smell

Dog olfaction serves as a unique biological baseline for all qualities. Dog detection is included in this review to demonstrate the unique biological sensitivity to the volatile organic chemicals found in human samples, but not to suggest that they should be used as diagnostic tools. The studies validate the human perceptibility of disease‐specific olfactory signatures and identify potential biomarkers can detect about 1000 different odorous chemicals present in a sample even at trace amounts. Their superior olfactory sensitivity and instantaneous functioning make them ideal biodetectors for a variety of chemicals [164]. Williams and Pembroke originally suggested using “sniffer dogs” to detect cancer in humans. They presented a case study wherein the owner’s pet dog assisted in the identification of malignant melanoma on the thigh. A similar study was detailed by Church and Williams twelve years later [165]. These studies depict how the dog owners’ lesions intrigued their innate curiosity, as they tried to bite, lick, and sniff them through their clothing. Upon removal of the malignant tumors, neither canine demonstrated an inclination in the sites. In a different study, it was shown that dogs of different breeds trained consistently can discriminate between samples that are malignant and those that are not by using a range of distinct odor sources [166, 167]. Pickel et al. used two canines to identify specimens of melanoma tissue placed on the skin of healthy people. The first dog showed signs of melanoma or probable melanoma in six of the seven individuals, which were later confirmed by pathological examination. The second dog identified four out of the seven patients, and the responses agreed with the first dog [168]. In another study, Willis et al. trained a group of six dogs of various breeds to detect bladder cancer by sniffing urine samples. The dogs successfully identified 41% of the cancer patients, with a 14% likelihood that the detections were made by chance. In another study, Willis et al. trained a group of six dogs of various breeds to detect bladder cancer by sniffing urine samples. The dogs successfully identified 41% of the cancer patients, with a 14% likelihood that the detections were made by chance [169]. In the study conducted by Horvath et al., a single dog was able to correctly identify ovarian cancer samples based on the odor profile of the tumor tissue. When tested in a blinded manner, the dog demonstrated remarkable accuracy, achieving 100% sensitivity and 97.5% specificity in distinguishing ovarian cancer samples from healthy tissues [170]. Cornu et al. assessed a dog’s capability to detect prostate cancer by analyzing urine samples. The dog demonstrated a sensitivity and specificity of 91% in this assessment [170, 171]. Four dogs were trained to identify lung cancer through reward‐based training. Exhaled breath samples were collected in glass tubes containing polypropylene fleece. Three subject groups with lung cancer, patients with COPD, and healthy donors were included [170, 171]. Four dogs were trained to identify lung cancer through reward‐based training. Exhaled breath samples were collected in glass tubes containing polypropylene fleece. Test results showed that the trained dogs achieved 71% overall sensitivity and 93% specificity [171, 172]. Buszewski et al. conducted the first study that compared canine detection of breath samples with GC‐TOF‐MS analysis to identify volatile biomarkers for lung cancer. Breath samples were collected in Tedlar bags and analyzed using Carboxen/PDMS fibers. The proposed method allowed for the detection of VOCs in breath at concentrations between 0.31 and 0.75 ppb [173]. Simultaneously, trained dogs were used to analyze breath samples from lung cancer patients and healthy individuals. Subjects exhaled through disposable polypropylene tubes with absorbent inserts. In a scent line up, four control samples and one randomly placed lung cancer sample were presented, with the dog handler blinded to the positions. The dogs correctly identified the lung cancer samples with 82.2% sensitivity and 82.4% specificity, with 17.8% false alerts for control samples. The difference between cancer and control indications was highly significant. Factor analysis and principal component analysis were used to evaluate the data, which showed that the concentrations of ethyl acetate and 2‐pentanone positively correlated with the dog’s indications, while acetonitrile, propanal, and 1‐propanol negatively correlated. This study provides further evidence that a trained dog can distinguish lung cancer patients from healthy controls by analyzing exhaled breath samples with a success rate higher than a chance. Our recent studies on the use of the trained dogs for detecting cancer‐associated VOCs have yielded promising results. Compared to the analytical techniques, canines were able to distinguish cancer odor samples from healthy controls rapidly, even after the samples have been stored for several weeks. Additionally, the dogs’ responses were binary in nature, which simplifies the interpretation of the findings relative to more complex analytical methods [174].

### 7.2. Breath Measurement in the ICU and During Surgery

Efforts are being made towards the development of reliable analytical techniques to understand the way different chemicals appear in the breath when mechanically ventilated. Every patient in the intensive care unit or undergoing surgery has trouble with breathing and is mechanically ventilated. Considering many patients undergoing surgery or residing in the intensive care unit rely on a ventilator for breathing, much work has gone into developing repeatable and trustworthy analytical procedures as well as comprehending how different substances come up in the breath when breathing on mechanical ventilation. A stark difference in substance concentrations may occur if dilution, contamination of the sample, or loss of analyte occurs during the sampling procedure. Sampling is done for a set length of time or for just a single breath. Microextraction techniques such as NTME and SPME are considered vital for preconcentrating breath indicators in clinical settings and subsequent lab‐based evaluation. Unlike SPME, NTME allows for enhanced identification limits through sample volume expansion and VOC biomarker adsorption direction variation. Both alone and in combination with mechanical breathing, these methods have proven successful in medical settings. Recent studies have shown that NTME is capable of being utilized in vivo for completely automated ionization and sampling operations [164, 175]. In seriously ill people, oxidative stress and inflammation were found to be the main causes of illness and organ dysfunction. Breath tests employing LPO indicators like pentane and ethane play a crucial role in clinically relevant breath studies. The number of transplant recipients receiving care in the intensive care unit is expanding. The most common causes include acute and chronic organ rejection and viral problems linked to immune suppression [175, 176]. With the advancements in science and technology in recent years, it is possible that soon VOC analysis in the exhaled air of critically ill patients will be used in clinical settings [14, 176].

### 7.3. Quantifying Oxidative Stress

Oxidative stress occurs when cells are harmed because of a chemical interaction with oxidative agents, such as free radicals produced from oxygen. These free radicals damage cell membranes, proteins, and genetic material by “oxidizing” them, much like the chemical mechanism that causes iron to rust. Activated granulocytes create elevated levels of reactive oxygen species (ROS), which can destroy any cellular structure. Typically, 2%–5% of the oxygen exchanged during respiration is utilized to generate ROS in mitochondria. Under healthy conditions, ROS activity is restricted to specific areas experiencing external assault or inflammation, and the body’s antioxidant defenses are well‐balanced. However, in some disease conditions, this equilibrium can be upset when antioxidant systems are overburdened or depleted, resulting in increased cellular damage [177].

The VOCs are generated during the process known as oxidative stress, which can be detected in exhaled breath, which provides a noninvasive means to assess oxidative stress. Free radicals, also known as chemical reaction catalysts, cause damage to the cell wall, genetic information, and proteins. A reliable biomarker of oxidative stress and airway inflammation is H_2_O_2_, which is detectable in EBC. In respiratory conditions such as asthma and COPD, increased levels of H_2_O_2_ in breath have been observed. Neutralization can be accomplished using H_2_O_2_, which can be detected in exhaled breath. H_2_O_2_ acts as a biomarker for oxidative stress. Oxidations produce alkanes by the conversion of PUFAs. This process results in the production of breath alkanes. Well‐established biomarkers of lipid peroxidation, specifically alkanes such as ethane and pentane, are observed while exhaled. This provides a conceptual framework for understanding the disease’s severity and probable repercussions. VOCs typically represent the outcomes of carbohydrate and lipid metabolism, oxidative stress, and the activity of cytochrome P450 liver enzymes within human cells [67]. In addition, VOCs also originate from both aerobic and anaerobic fermentation processes occurring in the gut microbiomes of bacterial communities. A multitude of VOCs, proteins, and peptides in water condensates, respiratory droplets, and exhaled breath aerosols have been identified as quantifiable biological indicators for diagnosing oxidative stress, inflammation, carcinogenic processes, and microbial infections. The measurement of breath pH, which represents the acid–base condition of the fluid lining the airways, is another complementary, noninvasive parameter in respiratory diagnostics. It has been demonstrated that the pH of breath condensate reduces airway inflammation in conditions like asthma, COPD, cystic fibrosis, and acute respiratory distress syndrome (ARDS). Significantly, this technique is authorized for use in children, which makes it useful for monitoring and early detection of pediatric respiratory conditions in situations where invasive sampling is not feasible. Given the importance of molecular oxygen in cellular metabolism, organisms have evolved complex defensive systems to preserve oxygen homeostasis. Aerobic cellular metabolic process is a four‐electron reduction of oxygen to water by virtue of mitochondrial oxidative phosphorylation being the major energy production route. Transport chain reaction is catalyzed by cytochrome; all four electrons move from oxygen to water (a four‐electron reduction). On the contrary, a small percentage of the molecular oxygen (1%–5%) features a step process of its reduction called monovalent leak [178].

Studies have shown that the concentrations of exhaled pentane and ethane correlate well with other LPO markers, such as malondialdehyde (MDA), thiobarbituric acid‐reactive substances (TBARS), and glutathione. In vitro investigations have also shown that ethane and pentane are produced when cell cultures are subjected to ROS. As a result, these molecules are used as both in vitro and in vivo indicators of lipid peroxidation. MDA and TBARS are two examples of breath indicators that are more sensitive than serum markers [96]. Antioxidants serve a key role in safeguarding organisms from the harmful effects of ROS by either reducing them to water or transforming them into less reactive nonoxygen‐based molecules. The interaction amongst ROS and antioxidant defenses is referred to as the oxidative stress condition. ROS has been linked to the development of an array of conditions, including cardiovascular, pulmonary, autoimmune, neurological, and inflammatory problems, as well as connective tissue and cancer. Additionally, ROS may contribute to the aging process. Lipid peroxidation, a well‐studied process, involves a chain reaction with separate initiation, propagation, and chain‐termination phases. In this process, ROS, commonly represented by the hydroxyl radical, take an allylic hydrogen atom from an unsaturated lipid, yielding a carbon‐centered radical and water. This radical goes through conjugation, peroxidation by molecular oxygen, and several following processes. The estimation of LPO entails measuring the stable molecules that come from this chain reaction. Hydrocarbons, conjugated dienes, aldehydes, isoprostanes, and lipid peroxides are some of the common examples of these stable products [179].

Lleberman et al. demonstrated that decreased triphosphopyridine nucleotide and pyrophosphate‐dependent LPO in rat liver microsomes was connected to the production of ethylene in the presence of cuprous ions. This system provides a model for the synthesis of ethylene in cells, which was attributed to the peroxidation of linolenic acid catalyzed by cuprous ions [180]. On the contrary, ethane was obtained by incubating linolenic acid with thioglycolic acid in the presence of a ferrous salt [181]. Together, these results show that oxidative damage in cells can take the form of detectable breath biomarkers, which are being used more and more in clinical diagnostics because they provide real‐time, noninvasive insights into disease processes.

## 8. Conclusions

In conclusion, the exploration of EBA presents a promising frontier in medical diagnostics and environmental monitoring. Identifying specific biomarkers associated with various diseases offers a noninvasive and convenient method of early detection and can revolutionize personalized healthcare. While diverse techniques for detecting biomarkers have been developed, each comes with its own set of advantages and limitations. The power of EBA lies in its accessibility and the wealth of information it can unveil about an individual’s health status. As we delve into assessing exposure to VOCs, understanding the parameters influencing their levels becomes imperative. From environmental factors to individual physiology, a comprehensive analysis is essential for accurate interpretation. Moreover, the ability to quantify oxidative stress through exhaled breath adds another layer of sophistication to this field, opening avenues for a comprehensive approach to health monitoring. Looking ahead, the future of EBA appears promising, with ongoing research likely to unveil novel biomarkers and enhance the precision of detection techniques. The integration of advanced technologies, artificial intelligence, and big data analytics holds the potential to refine our understanding of disease signatures within breath profiles. However, challenges persist, such as standardization of methodologies and addressing ethical considerations. The journey into EBA is marked by significant strides, offering a unique window into our health and environmental exposures. While we acknowledge the supremacy of this approach, we must remain vigilant in navigating its shortcomings. As we chart the course forward, the collaboration between researchers, healthcare professionals, and technology developers will be pivotal in unlocking the full potential of EBA for the benefit of global health and environmental sustainability.

**Nomenclature**
ANN: Artificial neural network
ANOVA: Analysis of variance
CDA: Canonical discriminant analysis
DA: Discriminant analysis
CEAS: Cavity‐enhanced absorption spectroscopy
CEPAS: Cavity‐enhanced photoacoustic spectroscopy
CRDS: Cavity ring‐down spectroscopy
CW‐ICOS: Continuous wave integrated cavity output spectroscopy
DFA: Discriminant factor analysis
DFB‐QCL: Distributed feedback‐quantum cascade laser
E‐nose: Electronic nose
EC‐ QCL: External cavity‐quantum cascade laser
FERS: Fiber‐enhanced Raman spectroscopy
FL: Fuzzy logic
FT‐IR: Fourier transform infrared spectroscopy
GC‐IMS: Gas chromatography–ion mobility spectroscopy
GC‐MS: Gas chromatography/mass spectrometry
GC‐TOF MS: Gas chromatography‐time of flight/MS
HPLC: High performance liquid chromatography
IMS: Ion mobility spectroscopy
KW‐test: Kruskal‒Wallis test
LAPS: Laser photoacoustic spectroscopy
LDA: Linear discriminant analysis
LIF: Laser‐induced fluorescence
LITES: Laser‐induced thermoacoustic spectroscopy
LPO: Lipid peroxidation
LR: Logistic regression
MC: Monte Carlo simulations
MCC‐IMS: Multicapillary column‐ion mobility spectrometry
MLR: Multiple linear regression
MWR‐test: Mann‒Whitney rank sum test
NMVS: Nanomaterial‐based VOC/gas sensor
NTME: Needle‐trap microextraction
OA‐ICOS: Off‐axis integrated cavity output spectroscopy
OF‐CEAS: Optical feedback cavity‐enhanced absorption spectroscopy
OFCS: Optical frequency comb spectroscopy
PCA: Principal component analysis
PNN: Probabilistic neural network
ppb: Parts per billion
ppm: Parts per million
ppt: Parts per trillion
PTR‐MS: Proton transfer reaction‐mass spectrometry
QCL: Quantum cascade laser
QDA: Quadratic discriminant analysis
QEPAS: Quartz‐enhanced photoacoustic spectroscopy
ROS: Reactive oxygen species
SERS: Surface‐enhanced Raman spectroscopy
SESI‐HRMS: Secondary electrospray ionization‐high resolution mass spectrometry
SESI‐MS: Secondary electrospray ionization
SFIT‐MS: Selective ion flow tube‐mass spectrometry
SPME‐GC/MS: Solid‐phase microextraction gas chromatography/mass spectrometry
SPME: Solid‐phase microextraction
*t* test: Student’s *t* test
TDLAS: Tunable diode laser absorption spectroscopy
*U* test: Mann‒Whitney *U* test
W‐test: Wilcoxon test
WDA: Weighted digital analysis

## Funding

The authors have nothing to report.

## Disclosure

All the investigators take responsibility for the integrity of the data and the accuracy of the data analysis. This includes all data from clinical trials regarding the use of EBA for disease detection (https://clinicaltrials.gov/).

## Ethics Statement

The authors have nothing to report.

## Consent

The authors have nothing to report.

## Conflicts of Interest

The authors declare no conflicts of interest.

## Data Availability

The datasets generated during and/or analyzed during the current study are available from the corresponding author upon reasonable request.
